# Multiscale Information Propagation in Emergent Functional Networks

**DOI:** 10.3390/e23101369

**Published:** 2021-10-19

**Authors:** Arsham Ghavasieh, Manlio De Domenico

**Affiliations:** 1 Department of Physics, University of Trento, Via Sommarive 5, 38123 Povo, Trento , Italy; 2 CoMuNe Laboratory, Fondazione Bruno Kessler, Via Sommarive 18, 38123 Povo, Trento, Italy

**Keywords:** information dynamics, multiscale analysis, networks entropy, network density matrix, fungal networks

## Abstract

Complex biological systems consist of large numbers of interconnected units, characterized by emergent properties such as collective computation. In spite of all the progress in the last decade, we still lack a deep understanding of how these properties arise from the coupling between the structure and dynamics. Here, we introduce the multiscale emergent functional state, which can be represented as a network where links encode the flow exchange between the nodes, calculated using diffusion processes on top of the network. We analyze the emergent functional state to study the distribution of the flow among components of 92 fungal networks, identifying their functional modules at different scales and, more importantly, demonstrating the importance of functional modules for the information content of networks, quantified in terms of network spectral entropy. Our results suggest that the topological complexity of fungal networks guarantees the existence of functional modules at different scales keeping the information entropy, and functional diversity, high.

## 1. Introduction

The underlying topology of complex systems is vital for their function, as it regulates the interactions between a system’s units and constrains the pathways for information flow which are necessary for the system to operate. However, a direct analysis of the network—by using its adjacency matrix representation—only provides information about the local interactions, the direct ones encoded in the adjacency matrix, thus missing the dynamic and multiscale nature of node–node communications. To shed light on communication between a system’s units at orders higher than one, i.e., beyond pairwise connections, a spectrum of possibilities are already available, from agent-based models where the maximum level of available details about the dynamics of units and the interactions between them are encapsulated into the equations, often aimed to calculate a desired quantity with high precision, to coupling general yet simple dynamical processes, such as random walks and diffusion, with the network to study the propagation of perturbations and transport of signals between the nodes, at first order approximation.

On the one hand, one can use random walk dynamics to gain insights about how information flow is locally trapped, allowing one, for instance, to uncover the functional mesoscale organization of classical [1], multilayer [2] and higher-order [3,4,5] networks, even across distinct scales [6,7,8,9]. Other exploration dynamics, such as walks, can be similarly used to capture the communicability between units and gain insights about the role of each node in exchanging information through the network [10,11,12,13]. On the other hand, one can employ higher-order models of the network structure [14,15] and study specific dynamics on the top of them, from synchronization [16,17,18] to social contagion [19,20,21], for instance.

Naturally, a rather holistic approach is required to capture the full information content of networks, their information dynamic and the multiscale communications between their units. In this work, we use diffusion processes to quantify the flow exchange between every pair of nodes and construct their emergent functional states, i.e., networks in which the links encode the information exchange between the nodes within a certain amount of time τ. As a case study of biological relevance, we consider a broad set of networks of fungi and slime molds, where structural links are obtained from cord conductances which, in turn, provide also a weight for pairwise connectivity (see [22] for details about the data set). This class of biological systems is peculiar in the way it stores and processes information: it has been shown that Physarum polycephalum (i.e., slime molds) grown on a rescaled map of Japan connects stations with a pattern resembling Tokyo’s rail system [23], and, even without a brain, they are able to externalize spatial memory to navigate complex environments [24], features which make them an exemplary model organism to understand the basic elements of computation in biological systems.

We show that such emergent functional states change with τ—acting as a tunable parameter encoding the propagation time of signals—while enabling one to analyze the multiscale nature of communications. Consequently, we show that analyzing the mesoscale organization of those emerging network states by means of an efficient algorithm, namely the Louvain method [25], it is possible to unravel the multiscale functional architecture of a system. In parallel, by using a recently developed statistical field theory of information dynamics [26], allowing for the definition of a suitable network density state [27,28] and multiscale analysis [29], we quantify the information content of networks in terms of their Von Neumann entropy, and show that the existence of functional modules is highly important for the macroscopic properties of systems.

Our results, when compared to randomized models of the fungal networks, suggest that their topological complexity plays a fundamental role in keeping their flow dynamics diverse, especially between middle- and long-range communication scales.

## 2. Materials and Methods

### 2.1. Propagator of Information Dynamics

The nodes of a complex network can be represented as canonical vectors $|x_{i}\rangle ,i=1,2,\cdots ,N$ —i.e., the equivalent of the vector ${\left(0,0,\cdots ,1,0,\cdots ,0\right)}^{T}$ in which only the *i*-th element is 1 and others are 0 in Dirac notation—, shaping a discrete and finite Euclidean space [30]. In this space, the pairwise connections between nodes can be encoded into an adjacency operator $\hat{W}$, where $\langle x_{j}|\hat{W}|x_{i}\rangle =W_{ij}$ provides the intensity of the link between node *i* and *j*. To capture flow dynamics within the network, one can assume the presence of a field |ϕ(τ)〉 on the top of its topology, free to evolve according to the diffusion equation

(1) $$\partial_{\tau }|\phi \left(\tau \right)\rangle =-\hat{L}|\phi \left(\tau \right)\rangle ,$$

$\hat{L}$ being the Laplacian operator $\langle x_{j}|\hat{L}|x_{i}\rangle =K_{i}\delta_{ij}-W_{ij}$, where $K_{i}=\sum_{j=1}^{N}W_{ij}$ is the strength defined as the summation of weights of links emanating from node *i*, and $\delta_{ij}$ is the Kronecker delta. The solution of the master equation in terms of the propagator $\hat{U}\left(\tau \right)=e^{-\tau \hat{L}}$ is given by

(2) $$|\phi \left(\tau \right)\rangle =\hat{U}\left(\tau \right)|\phi \left(0\right)\rangle .$$

Note that the framework is general enough to model the interactions between agents of a wide range of complex systems, either exactly or at first order approximation. As a general term, information exchange has been used to describe such interactions in systems that differ in physical attributes and nature of traveling signals between them, from electrical pulses in brain networks to news spreading among individuals. To quantify the propagation of the field with an initial value $\phi_{0}$ originating from node *i*, one can set the initial condition as $|\phi \left(0\right)\rangle =\phi_{o}|x_{i}\rangle$ and calculate the flow of the field from node *i* to any node *j* as $\phi_{0}\langle x_{j}|\hat{U}\left(\tau \right)|x_{i}\rangle$.

### 2.2. Emergent Functional States

Instead of focusing on the adjacency matrix, that provides a static and localized picture of interactions in the system, one can consider the propagator encoding pairwise flow exchange between the nodes at multiple scales, characterized by a tunable temporal parameter τ to span short-, middle- and long-range functional interactions between the nodes. To this aim, we define the emergent functional state as $\hat{\tilde{W}}\left(\tau \right)$ such that the interaction between node *i* and *j* follows

(3) $$\langle x_{j}|\hat{\tilde{W}}\left(\tau \right)|x_{i}\rangle =\phi_{0}U_{ij}\left(\tau \right)\left(1-\delta_{ij}\right),\;$$

where $\hat{\tilde{W}}\left(\tau \right)$ is, in practice, equivalent to the propagator rescaled by the constant $\phi_{0}$, excluding the diagonal entries $\langle x_{i}|\hat{U}\left(\tau \right)|x_{i}\rangle ,i=1,2,\cdots ,N$. The emergent functional state is, in fact, a multiresolution counterpart of the adjacency matrix, able to encode the interactions between the nodes, both locally and globally. Consequently, it allows for generalization of a range of concepts originally defined for analysis of structure, like degree distribution and modularity, beyond the limited local picture given by the adjacency matrix.

**Figure 1:**
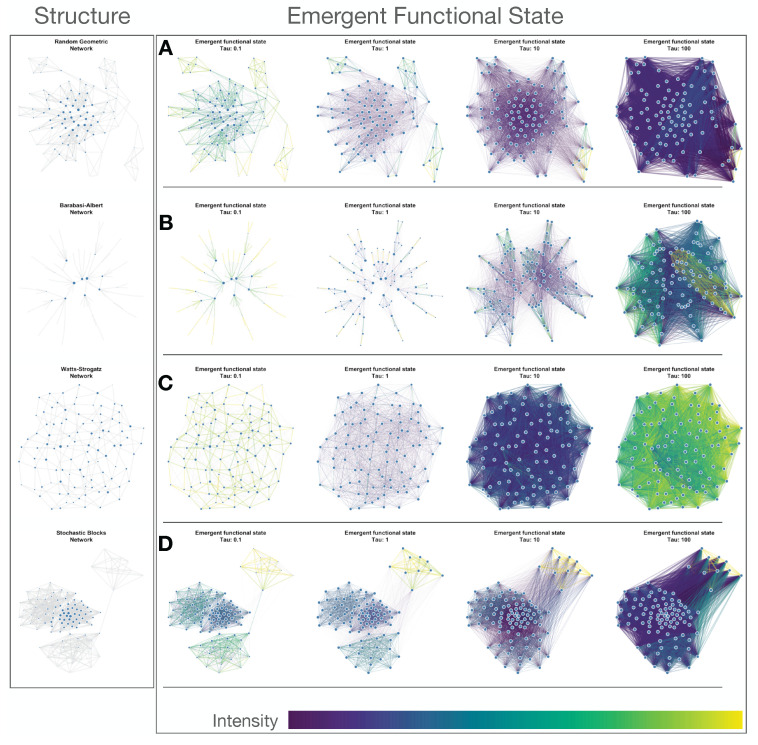
Emergent functional state. Emergent functional states associated with four different network types, at four different temporal scales, τ. On the left, the Structure column represents the considered synthetic networks exhibiting random geometric, Barabasi Albert, Watts Strogatz, and Stochastic Blocks topology. In front of each network type, its emergent functional state is visualized at four temporal scales: τ=0.1,1,10,100. Subplots show the emergent functional state of a (**A**) random geometric network with radius 0.18, (**B**) Barabasi Albert network with m=1, (**C**) Watts Strogatz network with mean degree of 4 and rewiring probability of 0.05, and (**D**) a Stochastic Blocks network having four communities with intra-community connectivity probability of $10^{-3}$ and inter-community connectivity probability of 0.5. The intensity of each link in the emergent functional state, characterized by the flow exchange between the nodes at the specific temporal scale, is colored from low (dark blue) to high (yellow).

**Figure 2:**
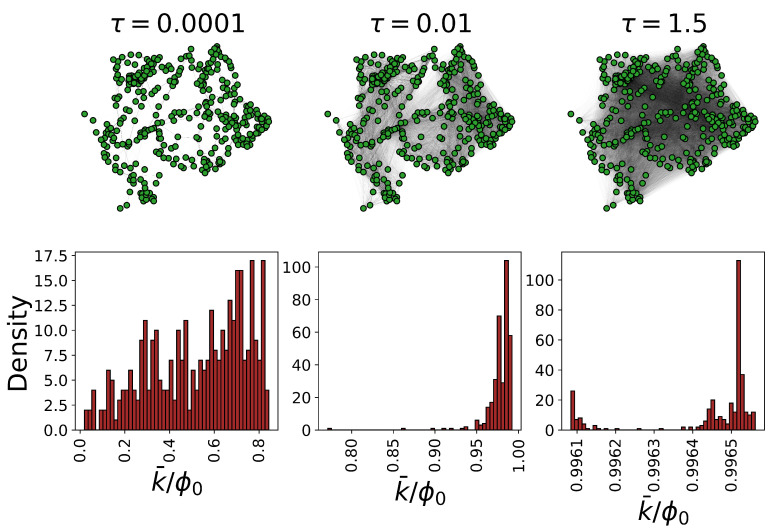
Connectivity distribution in emergent functional states of fungal networks. The emergent functional states corresponding to one realization of *Resinicium bicolor* networks, at 3 different scales (Markov time: τ=0.0001,0.01,1.5) are shown. Below each functional state, the corresponding strength distribution is reported. Tuning the temporal parameter from small to large values, one reaches a state where the functional state is fully entangled.

It is worth noting that, in the limit of the very small temporal scale, the propagator can be linearized as $\hat{U}\left(\tau \right)=e^{-\tau \hat{L}}\approx \hat{I}-\tau \hat{L}$, where $\hat{I}$ is the identity matrix, and the functional network adjacency reduces to the adjacency matrix ${\tilde{W}}_{ij}\left(\tau \right)=\phi_{0}\left(1-\delta_{ij}\right)U_{ij}\left(\tau \right)\approx \phi_{0}\left(1-\delta_{ij}\right)\left(\delta_{ij}-\tau K_{i}\delta_{ij}+\tau W_{ij}\right)=\phi_{0}\tau W_{ij}\left(1-\delta_{ij}\right)$, encoding local interactions between the nodes, while excluding self-loops. For clarity, a visualization of the emergent functional state corresponding to four synthetic networks is visualized in Figure 1. Moreover, for the emergent functional state $\hat{\tilde{W}}\left(\tau \right)$, we represent the strength of node *i* using small letter $k_{i}=\sum_{j=1}^{N}{\tilde{W}}_{ij}$. The strength distribution depends on the temporal scale of interactions τ (see Figure 2).

### 2.3. Emergent Functional Modules

A variety of algorithms have been developed to capture the communities in complex networks, including the Louvain method [25], a widely used and efficient one. Here, instead of analyzing the community structure of the underlying network, we focus on emergent functional states $\hat{\tilde{W}}\left(\tau \right)$. Each functional module consists of nodes that exchange a higher amount of flow between themselves than with the rest of the nodes. Naturally, the content of functional modules depends on the underlying network and the propagation time scale. When short range communications are considered, the number of communities is expected to be large, due to the large number of possible local neighborhoods. Conversely, at long range interactions, a smaller number of modules containing large numbers of nodes are expected to emerge.

It is worth mentioning that dynamical processes have been previously used to devise methods, like the Markov Stability framework, to identify communities. In the Markov Stability framework, a dynamical process like random walk at its equilibrium state is assumed on top of the network. In this case, meaningful communities are identified based on the probability that the random walker originating from these communities stays inside them over time.

Note that despite the similarity in using dynamical processes, our approach is fundamentally different. Firstly, the emergent functional state can be calculated for any general propagation process, regardless of the initial conditions. For instance, assume the fraction of the initial amount of the field $\phi_{0}$ on top of node *i* is given by $\frac{\phi_{i}}{\phi_{0}}$. In this case, this initial condition can be built into the propagator as follows :

(4) $$\hat{U}\left(\tau \right)=\hat{\Pi }e^{-\tau \hat{L}},$$

where $\Pi_{ij}=\frac{\phi_{i}}{\phi_{0}}\delta_{ij}$, generalizing Equation (3) to initial distributions other than uniform $\hat{\Pi }=\hat{I}$. Therefore, given that there is no constraint on the initial distribution, the modules appearing in the emergent functional state can be fundamentally different from the ones identified by the Markov Stability framework, which assumes the equilibrium distribution. Secondly, even if the initial distributions are taken to be the same, it is worth noting that by applying the Louvain method to the emergent functional states, one optimizes the modularity [31]. Of course, the result of such an algorithm can be coherent with the Markov Stability framework. However, the quality functions these two algorithms optimize are different and further studies are required to provide a comprehensive picture of their similarities and differences.

Finally, it is worth mentioning that our task here is to capture the functional communities in static networks. In other words, the propagation of signals in the network is assumed to happen significantly faster than the evolution of the links, leading to a separation of time-scales. Of course, even if the temporality of the network is not negligible, one can tune the parameter τ at scales below the network’s time-scale and perform the analysis presented in the study, directly. Otherwise, when the aim is to study larger τ, further theoretical development is required, generalizing the methods used to find evolving structural communities [32] to the functional ones.

### 2.4. Statistical Physics of Complex Information Dynamics

While approaches based on classical information theory and statistical physics have been widely used to study complex networks [33,34], they often rely on network descriptors such as degree and miss the full information content of interconnected systems. Conversely, inspired by developments in quantum thermodynamics, network density matrices have been proposed to study complex systems through the lens of Von Neumann entropy and without the information loss due to reducing the network to its descriptors [35]. Early definitions of the density matrix have been successfully applied to a range of problems, including the dimensionality reduction of multilayer networks [36], while they often were not physically interpretable and did not satisfy requirements such as sub-additivity.

Resolving the discussed issues related to the previous definitions, the network Gibbs state has been introduced [26,27,37] to quantify and compare the information content of complex networks. This framework has been successfully applied to a wide variety of problems in network science, from multilayer reducibility and its effect on the transport phenomena [28], to the human microbiome [27], the human brain [38,39], network robustness [40] and pan-viral interactomes [29].

In the following, we review the basics of this framework. The network Gibbs state is defined as

(5) $$\hat{\rho }\left(\tau \right)=\frac{\hat{U}\left(\tau \right)}{Tr[\hat{U}\left(\tau \right)]}.$$

Remarkably, it has been shown that the density matrix provides the smallest element to describe the flow dynamics in the system [26]. Furthermore, the density matrix is a superposition of an ensemble of operators, acting like streams directing the flow in the system. The mixedness of streams can be quantified using the Von Neumann entropy as

(6) $$S\left(\tau \right)=-Tr[\hat{\rho }\left(\tau \right)\log\hat{\rho }\left(\tau \right)],$$

which is a measure of diversity of flow in the system. Later in the text, we will show that the number of functional modules in fungal networks is proportional to the Von Neumann entropy.

### 2.5. Functional Diversity

Units of complex systems exhibit undeniable similarities in appearance, while they can take very different functional roles according to how they are positioned in the network. For instance, neurons belonging to different functional areas of the brain, like auditory and visual, are involved in different functions of the system [41], similar to individuals with different occupations in societies. The functional diversity of networks can be determined, from the perspective of information dynamics, in terms of the differences among the nodes as senders or receivers of information. It has been previously shown that the Von Neumann entropy is a proxy of functional diversity in the system [26], and the presence of topological complexity boosts the functional diversity of networks at middle to large propagation time scales τ. The functional diversity, in that study, is quantified in terms of the average cosine distance of the flow distribution vectors emanating from pairs of nodes.

Comparing the Von Neumann entropy of empirical networks with their configuration models—i.e., a model that generates a random network with the same degree distribution as the real network under study [42]—or types of randomized versions, has been used to investigate the benefits of topological complexity often observed in real systems for their functional diversity [26,39].

### 2.6. Rescaling the Temporal Scales across Networks

For a connected undirected network, let the eigenvalues of the Laplacian operator be $\lambda_{\ell },\ell =1,2,\cdots ,N$, ordered as $\lambda_{\ell }\leq \lambda_{\ell +1}$ and $\lambda_{1}=0$. Therefore, the eigenvalues of the propagator follows $e^{-\tau \lambda_{\ell }},\ell =1,2,\cdots ,N$. The diffusion process reaches equilibrium at τ→∞, where all the eigenvalues of the propagator decay except the one corresponding to the first eigenvalue of the Laplacian $e^{-\tau \lambda_{1}}=1$. The second eigenvalue of the Laplacian determines the diffusion time $\tau_{d}=1/\lambda_{2}$, that is the temporal scales at which the dynamics is close to equilibrium and the second eigenvalue of the propagator has decayed to $e^{-\tau_{d}\lambda_{2}}=\frac{1}{e}$.

The diffusion time is different from network to network, depending on the connectivity, number of nodes, topology, etc. To allow for comparison across networks, one can divide the propagation time scale τ by the diffusion time to obtain the rescaled temporal parameter $\tau /\tau_{d}$.

### 2.7. Fungal Networks

Here, we consider three species of fungi and slime molds: *Physarum polycephalum* (Pp), *Phanerochaete velutina* (Pv) and *Resinicium bicolor* (Rb). For each species, we analyze a certain number of networks to capture the average topological properties of the species. In these networks, the weight of the structural links is proportional to the cord conductance for pairwise interactions (see [22] for details about the data set). While the full data set consists of 270 networks, we focus on a subset of 92 networks with the number of nodes equal or smaller than 500, for computational convenience.

### 2.8. Randomized Networks

Often, properties of networks are understood in comparison with null models. The most famous null models for networks are Erdos-Renyi random models, networks of the same size and the same number of links as the original network while the links are randomly distributed between the nodes, and configuration models, where the degree distribution is fixed and the connections are randomized.

In the case of very sparse networks, like trees, the randomization of links leads a connected network to a network that is no longer connected, consisting of a number of disconnected sub-networks. Undoubtedly, the resulting disconnected network exhibits profoundly different information dynamics and, therefore, can only work as a null model that trivially and significantly differs from the original network. For instance, it has been shown that at large temporal scales, the Von Neumann entropy of networks can be approximated as the number of isolated components in disconnected networks [40], as an example of trivial differences between these null models and sparse networks.

The fungal networks under investigation here are both sparse and weighted. We exploit weights to generate suitable null models, defined as randomized-weight configuration model (RWCM), as follows: One fixes the position of links in the original network, while assigning random weights, from a uniform distribution, to the links. Finally, all the links are normalized by the summation of weights in the random network and multiplied by the summation of links in the original network. This normalization guarantees the overall weight strength of the null model equals the original network. Overall, RWCM keeps the network connected while randomizing the distribution of weights, providing null models for sparse weighted networks.

## 3. Results

### Functional Networks

We numerically calculate the emergent functional networks corresponding to all considered fungal networks, at different scales. When the temporal parameter is small, the flow would not penetrate into the network and the information exchange is limited to the nodes and their first neighbors. While, at large temporal scales, the functional network becomes a fully connected network where each node is connected to all others (see Figure 2 for an example). Note that the average degree (strength) of functional networks can be defined as

(7) $$\bar{k}=\frac{1}{N}\sum_{i,j=1}^{N}{\tilde{W}}_{ij},$$

which can be divided by $\phi_{0}$ to make it independent of the initial value of the field.

**Figure 3:**
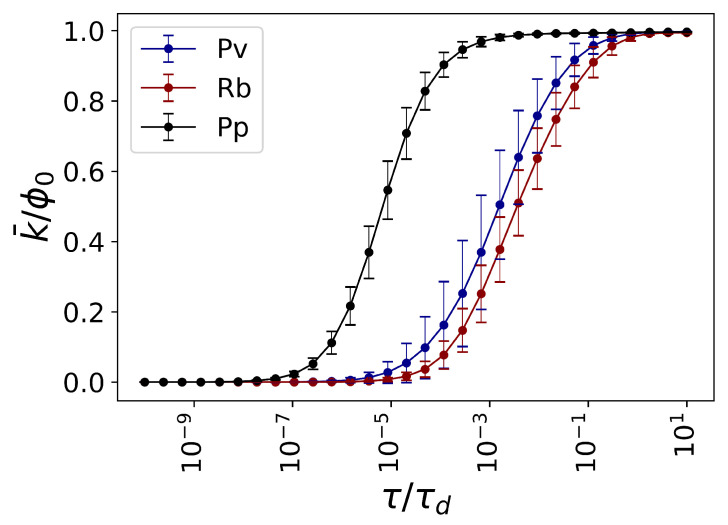
Average connectivity of emergent functional network states. The average strength of nodes $\bar{k}$ in the emergent functional state (see Methods) is plotted against the rescaled temporal parameter $\tau /\tau_{d}$ (see Methods). The relevant temporal scales for analysis of information dynamics depend on a variety of parameters, such as the number of nodes and their topology, reflected in the spectrum of the Laplacian matrix. During the transition from the extremely small rescaled temporal scale to the large rescaled temporal scale, it is observed that the average strength of the emergent functional state (see Methods) goes from zero, where no exchange flow is possible between the nodes, to $\phi_{0}$, where all the field originated from each node is completely distributed to the others. Here, we numerically observe that the relevant temporal scale for analysis of information dynamics in all three fungal species lays in $\tau /\tau_{d}\in \left[10^{-10},10\right]$.

We limit the temporal range to $\tau /\tau_{d}\in \left[10^{-10},10\right]$, to capture the full range of flow dynamics within all the fungal networks. Consequently, it is straightforward to show that the connectivity of functional networks increases with the rescaled temporal parameters (see Figure 3).

**Figure 4:**
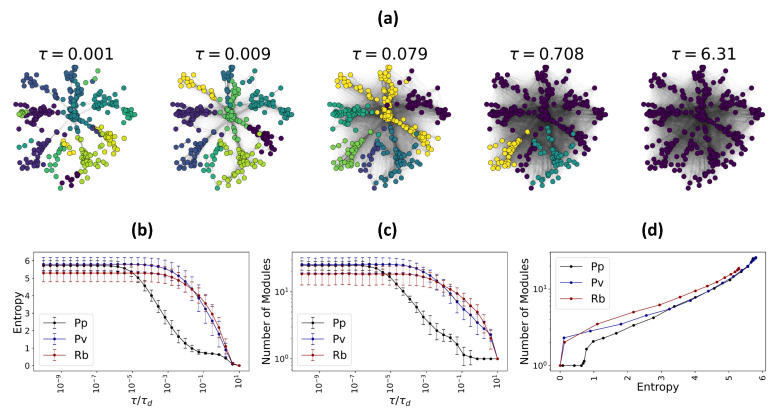
Multiscale functional fungal networks. Mesoscale organization of emergent functional states and the Von Neumann entropy of fungal networks, with species such as *Physarum polycephalum* (Pp), *Phanerochaete velutina* (Pv), and *Resinicium bicolor* (Rb) is illustrated. (**a**) Functional networks corresponding to a *Physarum polycephalum* network, at 5 different scales τ=0.001,0.009,0.079,0.708,6.31. The functional modules captured by the Louvain algorithm are colored differently. (**b**) Average entropy of all fungal networks considered in this study plotted as a function of rescaled temporal parameter $\tau /\tau_{d}$. (**c**) Average number of functional modules at each rescaled temporal parameter over all the fungal networks considered in this study. (**d**) Relation between the average number of functional modules with the Von Neumann entropy is shown.

Using the Louvain algorithm, we find the number of functional modules and their members in each functional fungal network ($\hat{\tilde{W}}$) at each temporal scale (see Figure 4). Furthermore, we calculate the entropy of the fungal networks ($\hat{W}$), to show that the number of functional modules scales is logarithmically proportional to the Von Neumann entropy of the network at each temporal scale.

**Figure 5:**
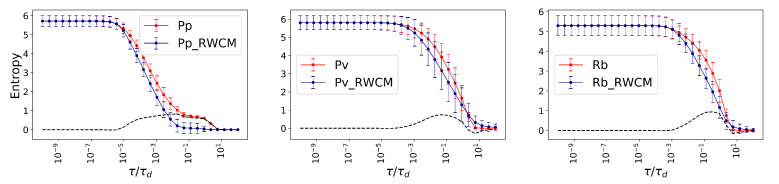
Entropy analysis. The average Von Neumann entropy for *Physarum polycephalum* (Pp), *Phanerochaete velutina* (Pv), and *Resinicium bicolor* (Rb) are shown as red lines. Their corresponding null models RWCM are plotted as blue lines. The Von Neumann entropy of the original networks and RWCM are indistinguishable when extremely small and large temporal scales are considered, while at the middle scales the Von Neumann entropy of original networks is often higher than the corresponding RWCM. The black dashed lines show the difference, to highlight the advantage of the real complex topology in keeping the functional diversity high.

Finally, we compare the Von Neumann entropy of the fungal networks with their RWCM (see Figure 5).

## 4. Discussion

Structural metrics are generally unable to capture different aspects of information flow within complex systems such as the fungal networks. Instead, a variety of methods have been developed, mostly based on coupling stochastic processes to the structural data and taking the higher order interactions into account, to study the functional interactions, transport phenomena, mesoscopic organization, and information content of networks [1,2,3,4,5,6,7,8,9,10,11,12,13,14,15,16,17,18,19,20,21].

In this article, we proposed a novel perspective in terms of emergent functional states, represented as networks where links encode the flow exchange between the nodes, according to diffusion processes. The emergent functional states can be calculated at different signal propagation time scales, describing short- to long-range functional interactions between the components of the system and providing a multiscale lens for systemic analysis. Consequently, we analyzed classical metrics of the emergent functional networks, such as degree distribution, encoding the total flow exchange of nodes within the networks, and the number and content of functional modules, obtained from feeding the emergent functional state as a network into the Louvain algorithm, which is based on modularity maximization. Moreover, we find numerical relations between the knowledge gained from the emergent functional networks and our information theoretic measures, to provide a broader picture. We show that the multiscale modular organization of the emergent functional states is a determinant of systems’ Von Neumann entropy, a proxy of the information content of networks [26]. Finally, we introduce the method of randomized-weight configuration models (RWCM), to obtain null models enabling us to compare the properties of empirical systems having highly sparse and weighted networks. We compare the fungal networks with their RWCM and show that they exhibit significantly larger Von Neumann entropy, indicating the efficiency of natural self-organization in providing structures that keep the flow dynamics diverse, even when long range communications are considered.

Potentially, artificial complex systems can be engineered to allow for optimal signaling properties, in terms of faster transport and increased functional diversity. The former has been explored theoretically and numerically and exploited to modify and design multiplex networks, such as airlines and urban transportation systems, with enhanced transport properties, using statistical physics of complex information dynamics [28]. The latter, however, is open to be explored, especially to investigate how different classes of networks compare to one another in terms of heterogeneity and diversity of diffusion pathways.

Our work provides a unifying picture of multiscale information dynamics and information content of fungal networks, further relating information dynamics to information-theoretic measures, a step towards an understanding of how collective computation occurs and emerges among interconnected biological units.

## Data Availability

The data are publicly available from the cited references.
